# Supplementary material for: ECD, a novel androgen receptor target promotes prostate cancer tumorigenesis by regulating glycolysis
Source: Oncogene. 2025 Sep 4;44(42):4058–74. doi: 10.1038/s41388-025-03559-x (PMC12518142; doi:10.1038/s41388-025-03559-x)
Supplement: Supplementary file 1 — Supplementary Figures and Tables [file 41388_2025_3559_MOESM1_ESM.pdf]

Table S1

List of ECD promoter mutants and the corresponding sequences deleted from TSS.

ECD promoter Mutants	Sequence deleted from TSS
CS-HPRM35342-LvPG04-01- site-1Δ	698 bp – 682 bp
CS-HPRM35342-LvPG04-02-site-2Δ	657 bp – 641bp
CS-HPRM35342-LvPG04-03-site-3Δ	640 bp – 624 bp
CS- HPRM35342-LvPG04-04-site-1,2Δ	698 bp – 682 bp, 657 bp – 641bp
CS-HPRM35342-LvPG04-05-site-1,3Δ	698 bp – 682 bp, 640 bp – 624 bp
CS-HPRM35342- LvPG04-06 - site-2,3Δ	657 bp – 641bp, 640 bp – 624 bp
CS-HPRM35342-LvPG04-07 - site-1,2,3Δ	698 bp – 682 bp, 657 bp – 641bp, 640 bp – 624bp

Table S2

List of real time qRT-PCR primers of human genes.

Primer Name	Forward Sequence	Reverse Sequence
LDHA	TTGACCTACGTGGCTTGGAAG	GGTAACGGAATCGGGCTGAAT
HKII	GAGCCACCACTCACCCTACT	ACCCAAAGCACACACCGGAAGTT
PKM2	CCACTTGCAATTATTTGAGGAA	GTGAGCAGACCTGCCAGACT
ECD	ACTTTGAAACACACGAACCTGGCG	TGATGCAGGTGTGTGCTAGTTCCT
18S	GCTTAATTTGACTCAACACGGGA	AGCTATCAATCTGTCAATCCTGTC
AR	TTGCAAGAGAGCTGCATCAGT	ACTGTGTGTGGAAATAGATGGGC

Table S3

Multivariate Cox's regression analysis of prostate cancer patient tumors with inclusion of cytoplasmic ECD expression
RR: Relative risk; CI: Confidence interval.

		Overall Survival		
	No. of Cases	RR	95%CI	p-value
**Cytoplasmic ECD Expression**				
negative/weak	315	1		
moderate/strong	241	1.689	1.085-2.629	0.02
**Tumour stage**				
postoperative pT2	386	1		
postoperative pT3	170	3.126	1.899-5.146	0
**Gleason**				
Score 2-6	192	1		
Score 7-10	364	2.415	1.247-4.675	0.009
**R-status**				
R0	396	1		
R1	157	1.666	1.038-2.675	0.035
**Age**				
≤60 Years	194	1		
>60 Years	362	0.971	0.934-1.011	0.151
**PreOP PSA**				
≤7.2ng/ml	279	1		
>7.2ng/ml	269	1.529	0.965-2.425	0.071

Table S4

Incidence of tumor formation in vector or ECD-overexpressing LNCaP xenograft model.

No. of Days	LNCaP Control group	ECD OE LNCaP Group
16	0 (0/26)/ 0%	6 (6/28)/ 21.4%
22	8 (8/26)/ 30.7%	20 (20/28)/ 71.4%
27	9 (9/26)/ 34.6%	23 (23/28)/ 82.1%
35	13 (13/26)/ 50%	26 (26/28)/ 92.8%

Fig. S1

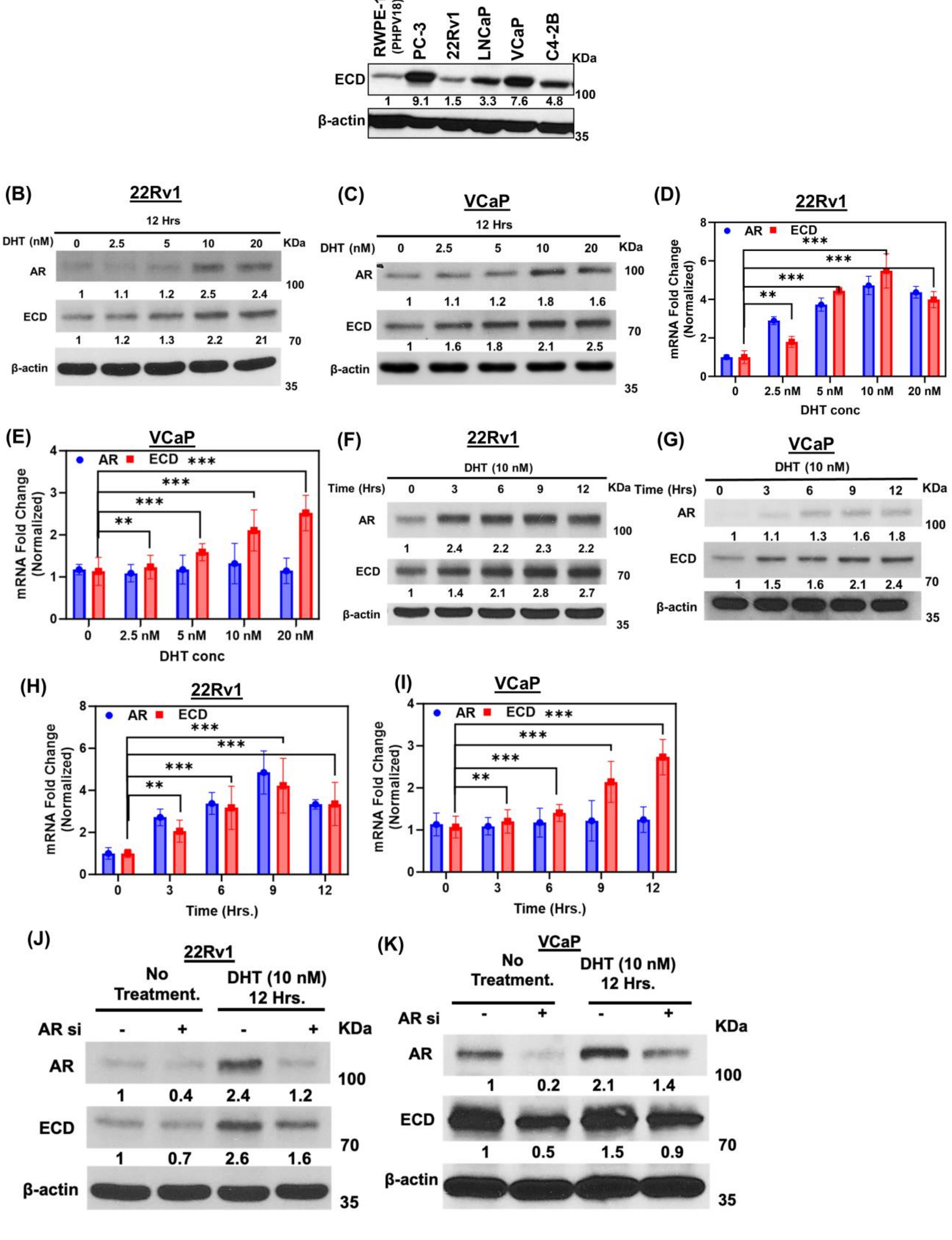
(A)
RWPE-1 (PHPV18)
PC-3
22Rv1
LNCaP
VCaP
C4-2B
KDa
ECD
1 9.1 1.5 3.3 7.6 4.8
100
β-actin
35
(B)
22Rv1
12 Hrs
DHT (nM) 0 2.5 5 10 20 KDa
AR
1 1.1 1.2 2.5 2.4
100
ECD
1 1.2 1.3 2.2 21
70
β-actin
35
(C)
VCaP
12 Hrs
DHT (nM) 0 2.5 5 10 20 KDa
AR
100
1 1.1 1.2 1.8 1.6
ECD
70
1 1.6 1.8 2.1 2.5
β-actin
35
(D)
22Rv1
AR ECD
mRNA Fold Change (Normalized)
0 2.5 nM 5 nM 10 nM 20 nM
DHT conc
(E)
VCaP
AR ECD
mRNA Fold Change (Normalized)
0 2.5 nM 5 nM 10 nM 20 nM
DHT conc
(F)
22Rv1
DHT (10 nM)
Time (Hrs) 0 3 6 9 12 KDa
AR
100
1 2.4 2.2 2.3 2.2
ECD
70
1 1.4 2.1 2.8 2.7
β-actin
35
(G)
VCaP
DHT (10 nM)
Time (Hrs) 0 3 6 9 12 KDa
AR
1 1.1 1.3 1.6 1.8
100
ECD
1 1.5 1.6 2.1 2.4
70
β-actin
35
(H)
22Rv1
AR ECD
mRNA Fold Change (Normalized)
0 3 6 9 12
Time (Hrs.)
(I)
VCaP
AR ECD
mRNA Fold Change (Normalized)
0 3 6 9 12
Time (Hrs.)
(J)
22Rv1
No Treatment.
DHT (10 nM) 12 Hrs.
AR si - + - + KDa
AR
1 0.4 2.4 1.2
100
ECD
1 0.7 2.6 1.6
70
β-actin
35
(K)
VCaP
No Treatment.
DHT (10 nM) 12 Hrs.
AR si - + - +
KDa
AR
1 0.2 2.1 1.4
100
ECD
1 0.5 1.5 0.9
70
β-actin
35

Fig. S1. Higher expression of ECD in PC cell lines; AR regulates ECD expression in 22Rv1 and VCaP cells. (A) WB showing expression level of ECD in indicated PC cell lines in comparison to HPV18 immortalized epithelial cells. **(B & C)** 22Rv1 and VCaP cells were cultured in steroid free conditions for 72 h. Cells were then treated with indicated concentrations of DHT for 12 h and immunoblotted with anti-ECD or anti-AR antibodies. β- actin was used as a loading control. **(D & E)** qRT-PCR was performed in the same samples; RNA was isolated by standard TRIzol phenol chloroform method. 18s rRNA was used for normalization. **(F-I)** Similar protocols as mentioned above were used to perform a time response with DHT. Western blotting **(F & G)** and qRT-PCR **(H & I)** were performed on cell lysates, as above. **(J & K)** 22Rv1 and VCaP cell lines were transfected with control siRNA or AR siRNA and cultured for 72 h. Cells were then treated with indicated concentrations of DHT for 12 h and then lysates were western blotted with anti-ECD or anti-AR antibodies. Numbers below the blots show the quantification of band intensities after normalizing with their respective loading control, β- actin using ImageJ software. Bar graphs represent mean ± SEM from three independent experiments, each done in triplicates. *Student's t*-test was used to calculate statistical significance *** $p < 0.001$, ** $p < 0.01$.

Fig. S2

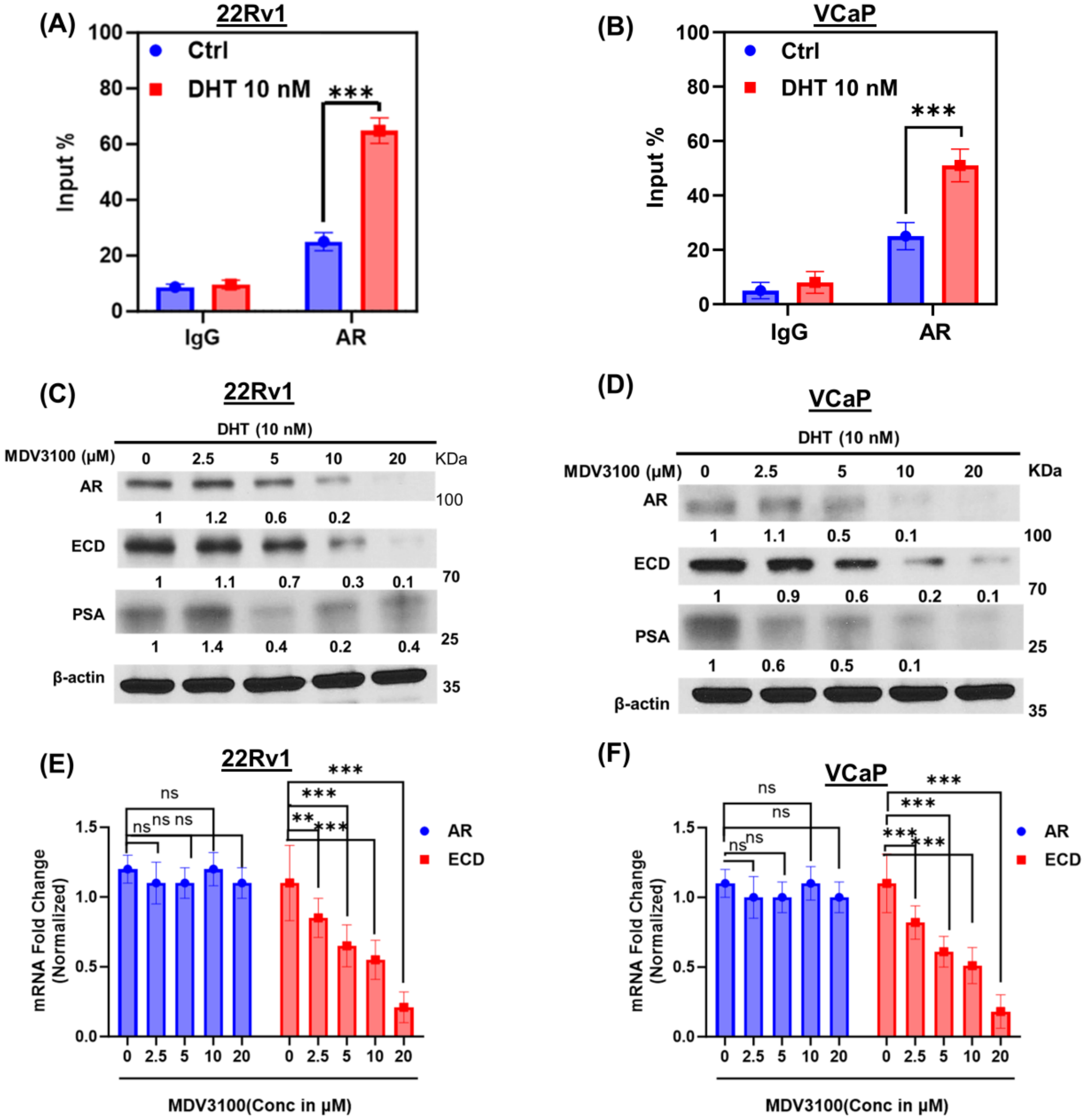

(A)
22Rv1
Ctrl
DHT 10 nM
***
Input %
IgG
AR
(B)
VCaP
Ctrl
DHT 10 nM
***
Input %
IgG
AR
(C)
22Rv1
DHT (10 nM)
MDV3100 (μM) 0 2.5 5 10 20 KDa
AR
100
1 1.2 0.6 0.2
ECD
1 1.1 0.7 0.3 0.1
70
PSA
25
1 1.4 0.4 0.2 0.4
β-actin
35
(D)
VCaP
DHT (10 nM)
MDV3100 (μM) 0 2.5 5 10 20 KDa
AR
1 1.1 0.5 0.1
100
ECD
1 0.9 0.6 0.2 0.1
70
PSA
25
1 0.6 0.5 0.1
β-actin
35
(E)
22Rv1
mRNA Fold Change (Normalized)
AR
ECD
MDV3100(Conc in μM)
(F)
VCaP
mRNA Fold Change (Normalized)
AR
ECD
MDV3100(Conc in μM)

Fig. S2. Increased AR occupancy on ECD promoter upon androgen treatment; enzalutamide treatment decreases ECD expression. (A & B) Indicated cells were cultured in steroid-free conditions for 3 days, followed by dihydrotestosterone (DHT) treatment for 3 h. Cells were cross-linked with formaldehyde and chromatin was immunoprecipitated with antibody against AR or IgG, used as a non-binding Control. Immunoprecipitated DNA was quantified with qRT-PCR for specific binding sites at ECD promoter using primers encompassing all three sites -712 to -565 from transcription start site. Histograms show relative pull-down enrichment of ECD promoter by IgG and AR from three independent experiments. Data represent mean ± SEM. *Student's t*-test was performed to calculate statistical significance *** $p < 0.001$. **(C & D**) Indicated cell lines were cultured in steroid-free medium for 72 h, cells were then treated with 10 nM DHT, followed by treatment with indicated concentration of MDV3100 (enzalutamide) for another 48 h. Lysates were collected and western blotted with indicated antibodies. Numbers below the blots show the quantification of band intensities after normalizing with their respective loading control, β- actin in comparison with control samples using ImageJ software. **(E & F)** qRT-PCR was performed in the same samples where the RNA was isolated by standard TRIzol phenol chloroform method. 18s rRNA was used for normalization. Bar graphs represent fold change ± SEM in AR and ECD mRNA in 22Rv1 **(E)** and VCaP cells **(F)** with respect to vehicle treatment from three independent experiments. *Student's t*-test was performed to calculate statistical significance *** $p < 0.001$, ** $p < 0.01$**.**

Fig. S3

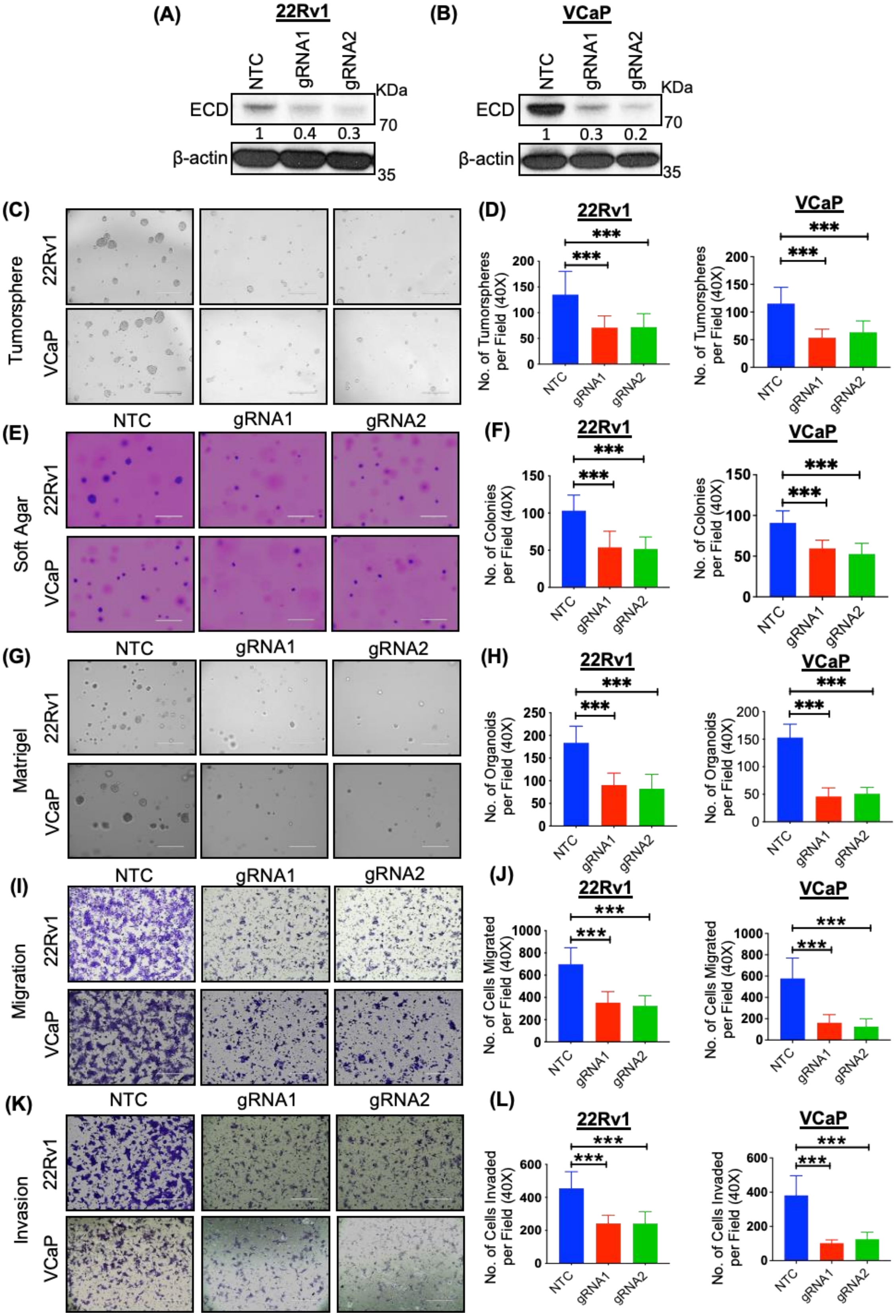

(A)
22Rv1
NTC
gRNA1
gRNA2
KDa
ECD
1
0.4
0.3
70
β-actin
35
(B)
VCaP
NTC
gRNA1
gRNA2
KDa
ECD
1
0.3
0.2
70
β-actin
35
(C)
Tumorsphere
22Rv1
VCaP
(D)
22Rv1
VCaP
No. of Tumorspheres per Field (40X)
***
NTC
gRNA1
gRNA2
(E)
Soft Agar
NTC
gRNA1
gRNA2
22Rv1
VCaP
(F)
22Rv1
VCaP
No. of Colonies per Field (40X)
(G)
Matrigel
NTC
gRNA1
gRNA2
22Rv1
VCaP
(H)
22Rv1
VCaP
No. of Organoids per Field (40X)
(I)
Migration
NTC
gRNA1
gRNA2
22Rv1
VCaP
(J)
22Rv1
VCaP
No. of Cells Migrated per Field (40X)
(K)
Invasion
NTC
gRNA1
gRNA2
22Rv1
VCaP
(L)
22Rv1
VCaP
No. of Cells Invaded per Field (40X)

Fig. S3. ECD depletion diminishes oncogenic traits in 22Rv1 and VCaP prostate cancer cells. Indicated cells expressing doxycycline (Dox) regulated Cas9 and guide RNAs targeting either ECD (gRNA1 & 2) or non-targeting control (NTC) were cultured in the presence of Dox (1 μg/ml) for 96 h to deplete ECD. **(A & B)** Western blot depicting expression of ECD in 22Rv1 **(A)** and VCaP **(B)** post Dox (1 μg/ml) treatment for 96 h. Numbers below the blot show the quantification of band intensities after normalizing with their respective loading control β-actin in comparison with NTC, using ImageJ software. Representative images and histograms depicting tumorsphere **(C & D),** soft agar colony **(E & F**), Matrigel organoid formation **(G & H),** migration **(I & J),** and invasion **(K & L),** of control and ECD depleted cells. The representative images of tumorspheres, soft agar colonies, Matrigel organoids, migration and invasion are at x100 magnification, scale bar, 400 μm. The Bar graphs represent mean values ± SEM of three independent experiments from x40 magnification images, each done in triplicates. *Student's t*-test was used to calculate statistical significance *** $p < 0.001$.

Fig. S4

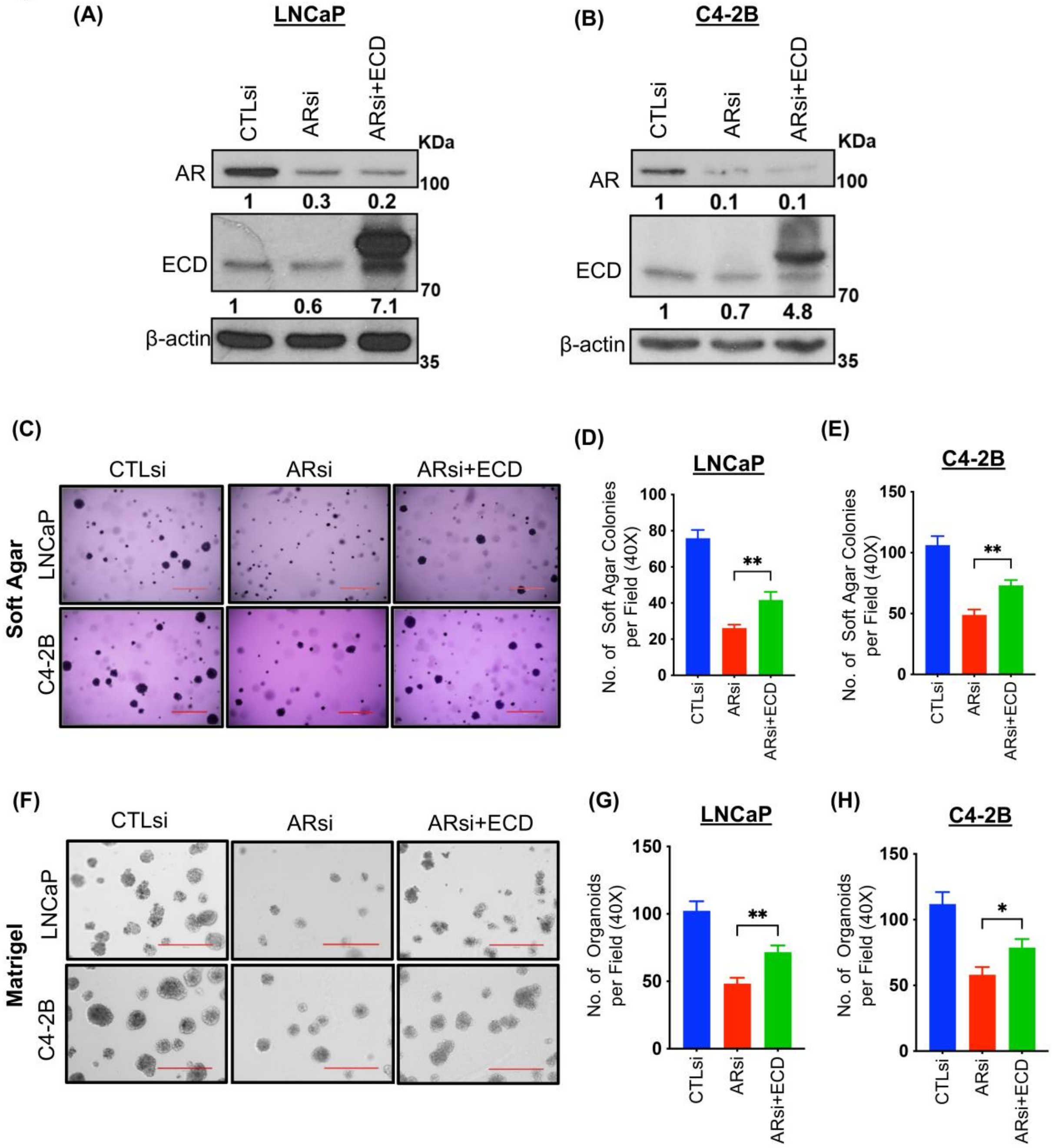

(A)
LNCaP
CTLsi
ARsi
ARsi+ECD
KDa
AR
100
1
0.3
0.2
ECD
70
1
0.6
7.1
β-actin
35
(B)
C4-2B
CTLsi
ARsi
ARsi+ECD
KDa
AR
100
1
0.1
0.1
ECD
70
1
0.7
4.8
β-actin
35
(C)
CTLsi
ARsi
ARsi+ECD
Soft Agar
LNCaP
C4-2B
(D)
LNCaP
No. of Soft Agar Colonies per Field (40X)
**
CTLsi
ARsi
ARsi+ECD
(E)
C4-2B
No. of Soft Agar Colonies per Field (40X)
**
CTLsi
ARsi
ARsi+ECD
(F)
CTLsi
ARsi
ARsi+ECD
Matrigel
LNCaP
C4-2B
(G)
LNCaP
No. of Organoids per Field (40X)
**
CTLsi
ARsi
ARsi+ECD
(H)
C4-2B
No. of Organoids per Field (40X)
*
CTLsi
ARsi
ARsi+ECD

Fig. S4. ECD overexpression partially rescues the effect of AR knockdown on oncogenic traits of PC cells. LNCaP and C4-2B cells were transfected with AR siRNA with or without ECD overexpression. Cell lysates were collected 96 h post transfection. **(A & B)** Western blot depicting expression of AR and ECD post AR knockdown. Numbers below the blot show the quantification of band intensities after normalizing with their respective loading control, β- actin in comparison with vector control using ImageJ software. Representative images and histograms depicting soft agar colony **(C-E)** and Matrigel organoid formation **(F-H),** of LNCaP and C4-2B post AR knockdown with or without ECD overexpression. The representative images of soft agar colonies and Matrigel organoids are at x100 magnification, scale bar, 400 µm. Bar graphs show mean values ± SEM of three experiments done in triplicates from x40 magnification images. *Student's t*-test was used to calculate statistical significance ** $p < 0.01$, * $p < 0.05$.

Fig. S5

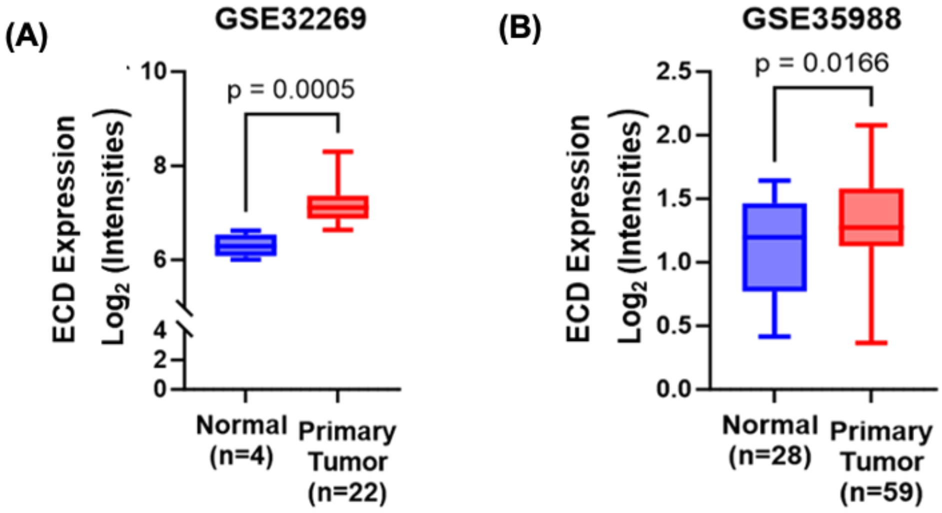

Fig. S5. PC tissue specimens show higher expression of *ECD* mRNA. Box plot showing mRNA expression of *ECD* from **(A)** 4 normal and 22 tumor samples from cohort 1 (GSE32269) [1] and **(B)** 28 normal and 59 tumor samples from another independent cohort (GSE35988) [2]. Two publicly available datasets were analyzed for *ECD* mRNA expression. A *paired t*-test was used to compare expression in normal versus cancer samples.

Fig. S6

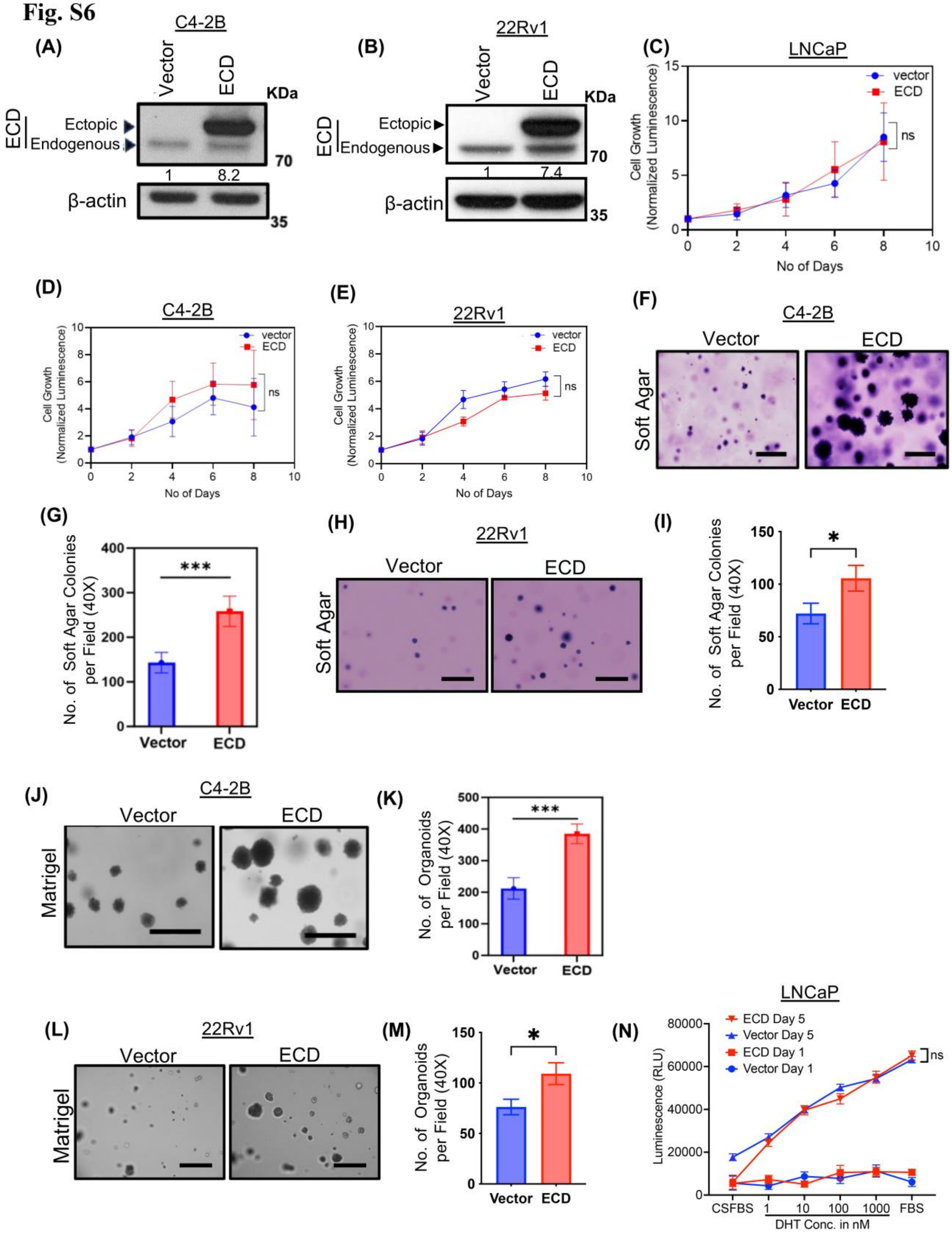
(A)
C4-2B
Vector
ECD
KDa
ECD
Ectopic
Endogenous
70
1
8.2
β-actin
35
(B)
22Rv1
Vector
ECD
KDa
ECD
Ectopic
Endogenous
70
1
7.4
β-actin
35
(C)
LNCaP
vector
ECD
ns
Cell Growth (Normalized Luminescence)
No of Days
(D)
C4-2B
vector
ECD
ns
Cell Growth (Normalized Luminescence)
No of Days
(E)
22Rv1
vector
ECD
ns
Cell Growth (Normalized Luminescence)
No of Days
(F)
C4-2B
Vector
ECD
Soft Agar
(G)
No. of Soft Agar Colonies per Field (40X)
***
Vector
ECD
(H)
22Rv1
Vector
ECD
Soft Agar
(I)
No. of Soft Agar Colonies per Field (40X)
*
Vector
ECD
(J)
C4-2B
Vector
ECD
Matrigel
(K)
No. of Organoids per Field (40X)
***
Vector
ECD
(L)
22Rv1
Vector
ECD
Matrigel
(M)
No. of Organoids per Field (40X)
*
Vector
ECD
(N)
LNCaP
ECD Day 5
Vector Day 5
ECD Day 1
Vector Day 1
ns
Luminescence (RLU)
CSFBS
1
10
100
1000
FBS
DHT Conc. in nM

Fig. S6. ECD-overexpressing PC cells exhibit enhanced anchorage independence and Matrigel three-dimensional (3D) growth but show no effects in 2D proliferation rate or androgen response. (A & B) Western blot shows overexpression of ECD in C4-2B **(A)** and 22Rv1 **(B)** cell lines. Numbers below the blot shows the quantification of band intensities after normalizing with their respective loading control, β- actin in comparison with vector cells using ImageJ software. **(C-E)** Cell proliferation curves showing proliferation rate of LNCaP **(C),** C4-2B **(D)** and 22Rv1 **(E)** cells overexpressing vector or ECD. Assays were performed using Cell-Titer-Glo**.** Luminescence values were plotted against number of days and normalized to day 1. Data represent mean ± SEM with two-way ANOVA test. $n = 3$; ns, $P > 0.05$. **(F & H)** Representative images in x100 magnification (scale bar, 400 μm) depict soft agar colony formation abilities upon overexpression of ECD in C4-2B **(F)** and 22Rv1 **(H)** cells. Images were taken after fixing and staining the colonies with 0.05% crystal violet. **(G & I)** Bars depict the mean ± SEM of soft agar colonies using images at x40 magnification in vector and ECD overexpressing C4-2B **(G)** and 22Rv1 **(I)** cells. Data represent three independent experiments, each done in triplicates. ***p < 0.001, *p < 0.05. *Student's t*-test was used to calculate statistical significance. **(J & L)** Representative images in x100 magnification (scale bar, 400 μm) depict 3D colony formation abilities of C4-2B **(J)** and 22Rv1 **(L)** cells in Matrigel upon overexpression of ECD. **(K & M)** Bar graphs show mean ± SEM of 3D colonies number quantified using images at x40 magnification in vector and ECD overexpressing C4-2B **(K)** and 22Rv1 **(M)** cells. Data represent three independent experiments, each done in triplicates. ***p < 0.001, *p < 0.05. *Student*'s *t*-test was used to calculate statistical significance. **(N)** LNCaP cells overexpressing vector or ECD were cultured in charcoal-stripped serum containing phenol red free medium for 72 h. Cells were then treated with indicated concentrations of DHT. Growth Assays were performed using Cell-Titer-Glo**.** Luminescence

values were plotted for both Day 1 and Day 5. Data represent three independent experiments, each done in triplicates. Two-way ANOVA (mixed model) was used to calculate statistical significance, ns, $p > 0.05$.

Fig. S7

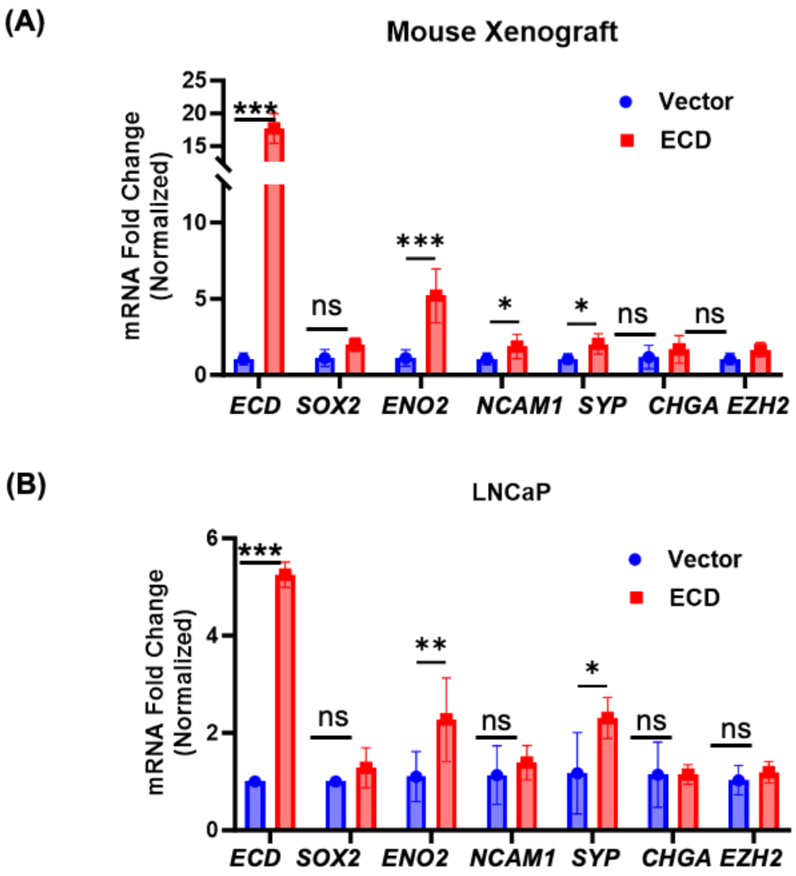

Fig. S7. qRT-PCR validation of neuroendocrine PC markers from transcriptomic analysis of ECD overexpressing LNCaP xenograft tumors. (A & B) qRT-PCR analysis of indicated neuroendocrine (NE) signature genes of vector or ECD overexpressing LNCaP xenograft tumors **(A),** and vector or ECD overexpressing LNCaP cells **(B).** 18s rRNA was used for normalization. mRNA quantitation data represent mean ± SEM with two-tailed unpaired *t*-test. $n = 3$; ns, $P > 0.05$, **, $P < 0.01$; ***, $P < 0.001$.

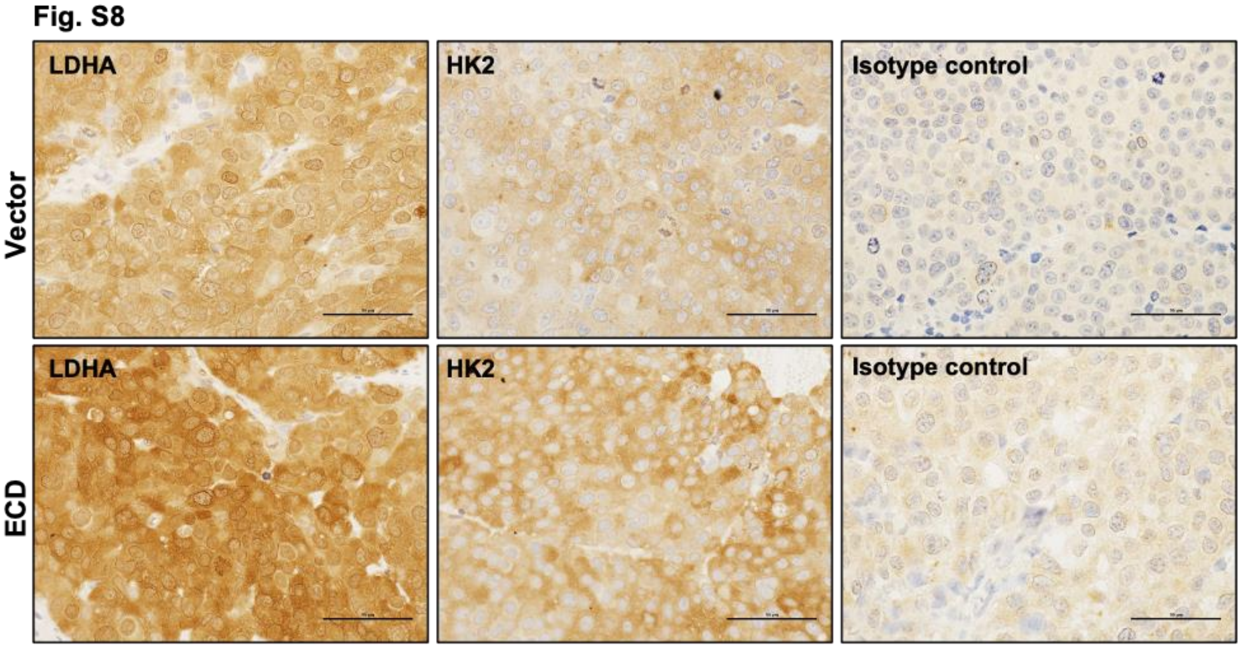

Fig. S8. Increased expression of glycolytic enzymes, LDHA and HK2 in ECD overexpressing LNCaP tumors. IHC analysis of paraffin embedded sections of LNCaP xenografts tumors from vector or ECD-overexpressing groups with anti-LDHA or anti- HK2 antibodies. Rabbit IgG was used as isotype control for the staining. Representative images x400 magnification (scale bar, 50 μm).

Fig. S9

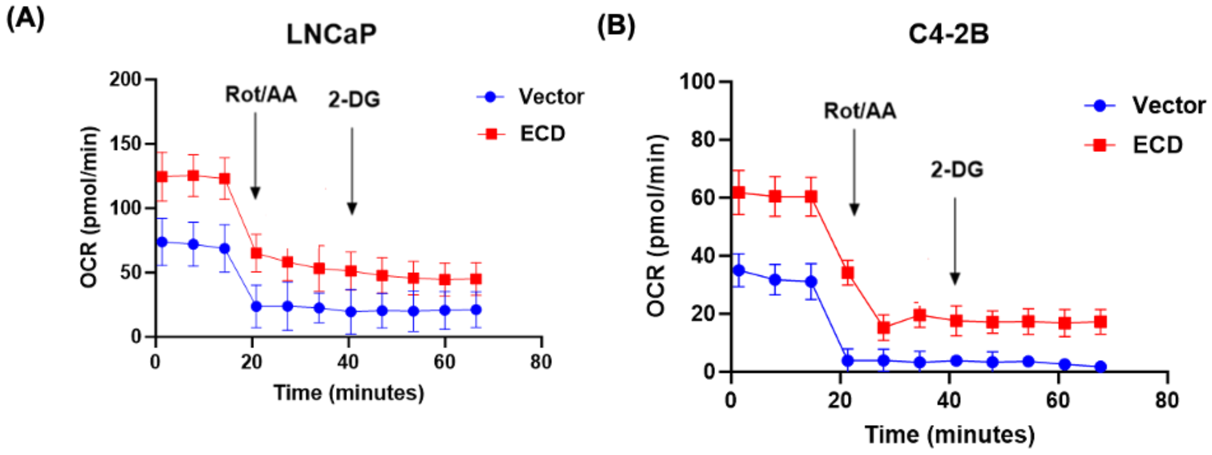

Fig. S9. Seahorse Glycolytic Rate assay in prostate cancer cell lines overexpressing vector or ECD. (A & B) Oxygen consumption rates (OCR) at various time points followed upon injections of Rot/AA (0.5 µM), and 2-DG (50 mM) in vector or ECD-OE cells.

Fig. S10

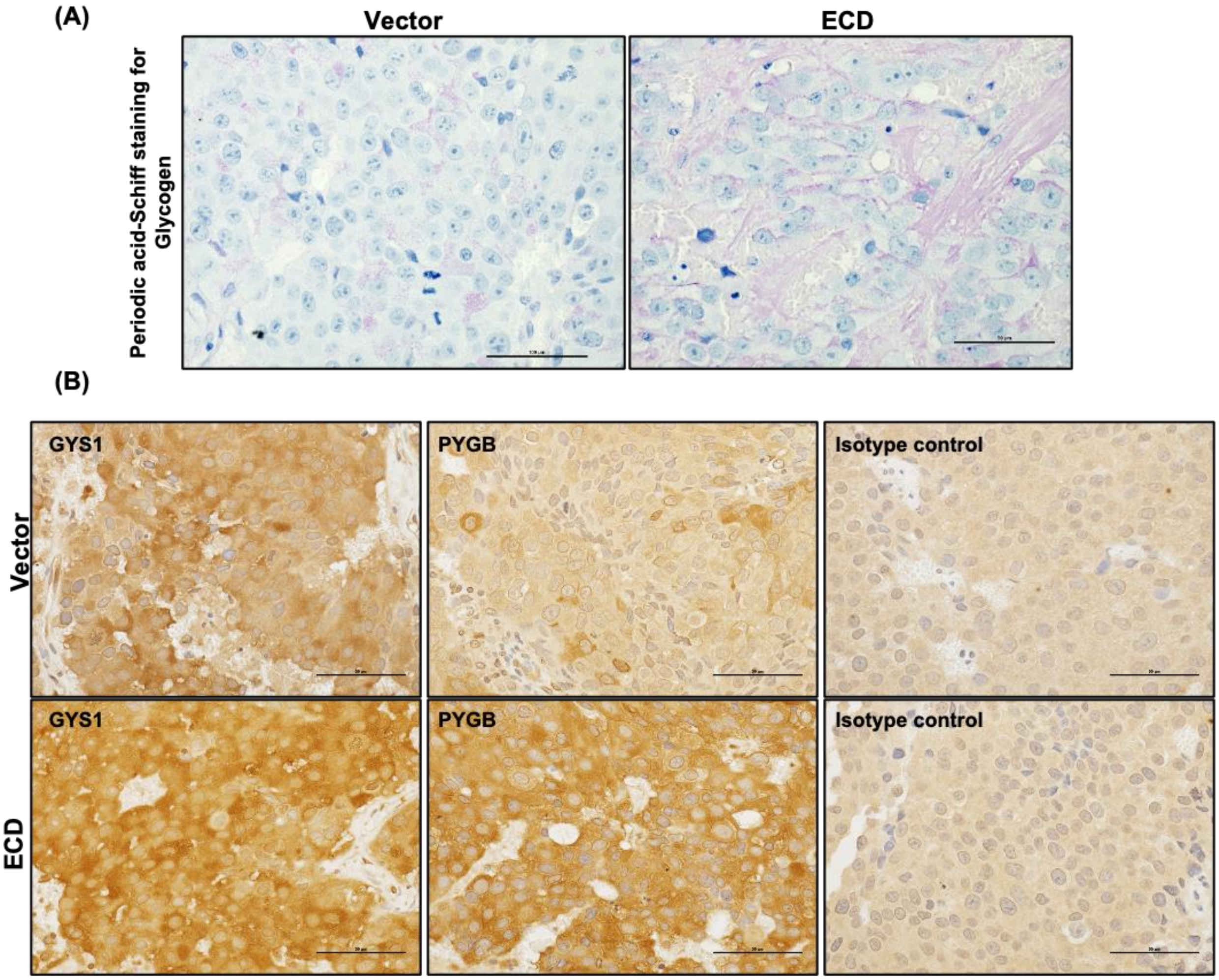

Fig. S10. Enhanced glycogenesis and glycogen metabolism in ECD overexpressing LNCaP tumors. (A) Periodic acid-Schiff staining shows increased glycogen deposition represented by magenta staining in ECD overexpressing tumors compared to vector expressing tumors. (**B**) IHC analysis of paraffin embedded sections of LNCaP xenografts tumors from vector or ECD-overexpressing groups stained with antibodies against glycogen synthase 1 (GYS1) and glycogen phosphorylase, brain (PYGB). Rabbit IgG was used as isotype control for the staining. Representative images are at x400 magnification (scale bar, 50 μm).

References:

1. Cai C, Wang H, He HH, Chen S, He L, Ma F *et al*. ERG induces androgen receptor-mediated regulation of SOX9 in prostate cancer. *J Clin Invest* 2013; 123: 1109-1122.

2. Grasso CS, Wu YM, Robinson DR, Cao X, Dhanasekaran SM, Khan AP *et al*. The mutational landscape of lethal castration-resistant prostate cancer. *Nature* 2012; 487: 239-243.
